# Engineering Macromolecular Trafficking Into the Citrus Vasculature

**DOI:** 10.3389/fpls.2022.818046

**Published:** 2022-02-01

**Authors:** Berenice Calderón-Pérez, José Abrahán Ramírez-Pool, Leandro Alberto Núñez-Muñoz, Brenda Yazmín Vargas-Hernández, Abel Camacho-Romero, Mariana Lara-Villamar, Domingo Jiménez-López, Beatriz Xoconostle-Cázares, Roberto Ruiz-Medrano

**Affiliations:** Departamento de Biotecnología y Bioingeniería, Centro de Investigación y de Estudios Avanzados, Mexico City, Mexico

**Keywords:** systemic signaling, macromolecules long-distance transport, supracellular proteins, vasculature, Citrus, antimicrobial proteins, plant defense

## Abstract

The plant vasculature is a central organ for long-distance transport of nutrients and signaling molecules that coordinate vegetative and reproductive processes, and adaptation response mechanisms to biotic and abiotic stress. In angiosperms, the sieve elements are devoid of nuclei, thus depending on the companion cells for the synthesis of RNA and proteins, which constitute some of the systemic signals that coordinate these processes. Massive analysis approaches have identified proteins and RNAs that could function as long-range signals in the phloem translocation stream. The selective translocation of such molecules could occur as ribonucleoprotein complexes. A key molecule facilitating this movement in Cucurbitaceae is the phloem protein CmPP16, which can facilitate the movement of RNA and other proteins into the sieve tube. The CmPP16 ortholog in Citrus CsPP16 was characterized *in silico* to determine its potential capacity to associate with other mobile proteins and its enrichment in the vascular tissue. The systemic nature of CsPP16 was approached by evaluating its capacity to provide phloem-mobile properties to antimicrobial peptides (AMPs), important in the innate immune defense. The engineering of macromolecular trafficking in the vasculature demonstrated the capacity to mobilize translationally fused peptides into the phloem stream for long-distance transport. The translocation into the phloem of AMPs could mitigate the growth of *Candidatus* Liberibacter asiaticus, with important implications for crop defense; this system also opens the possibility of translocating other molecules to modulate traits, such as plant growth, defense, and plant productivity.

## Introduction

Plants synthesize mobile informational macromolecules, such as RNA (small, long non-coding, and messenger RNAs), and proteins that travel to distant tissues to coordinate developmental processes and responses to both biotic and abiotic stress (Ruiz-Medrano et al., 2001, 2004; Ham and Lucas, 2017; Kehr and Kragler, 2018). The translocation of these signals occurs in the sieve elements of the plant vasculature, which also carries sugars, hormones, amino acids, sugar alcohols, and other organic compounds. The direction of such translocation occurs from source to sink tissues (Caño-Delgado et al., 2010; Campbell and Turner, 2017). In higher plants, the vasculature differentiates from the cambium cells to xylem tissue and transports water and mineral salts. The cambium meristem differentiates in the opposite direction to produce the phloem tissue. This conducting organ is formed of enucleated sieve elements (SEs) and companion cells (CCs), which have an elongated shape, high cytoplasmic density, and are tightly connected to the sieve tube through specialized plasmodesmata (Miyashima et al., 2013; Ohashi-Ito and Fukuda, 2014; Ruonala et al., 2017). CC transcribes RNAs and synthesizes proteins that are translocated selectively to the SE. Some travel to distant tissues as proteins or ribonucleoprotein complexes and function in a non-cell-autonomous manner; however, the significance of mobile RNA trafficking is not clear in most cases (Ruiz-Medrano et al., 2001, 2004; Notaguchi, 2015; Saplaoura and Kragler, 2016; Ham and Lucas, 2017).

Hundreds of RNAs and proteins can travel across a graft union and are thought to exert their function on cellular targets, triggering adaptive responses (Haywood et al., 2005; Banerjee et al., 2006; Toscano-Morales et al., 2014). Massive systemic movement of RNAs and proteins occurs between parasitic plant and their hosts as well, including microRNAs that could downregulate the host defense response (Kim et al., 2014; Westwood and Kim, 2017; Subhankar et al., 2021).

Among the physiological functions of phloem-mobile RNAs and proteins are the regulation of flowering, tuberization, and phosphate sensing; indeed, signals synthesized in photosynthetic tissues travel to distant sink tissues, such as the apical meristem of the shoot for flowering and the root initials for tuberization (Corbesier et al., 2007; Lin et al., 2007; Pant et al., 2008; Navarro et al., 2011). A more recent example is the basic Helix–Loop–Helix (bHLH) transcription factor *Cucumis sativus* Irregular Vasculature Patterning that functions in the development of the vasculature and the resistance to downy mildew in cucumber (Yan et al., 2020). Furthermore, phloem-mobile RNAs induced by phosphate deficiency include long non-coding RNAs (Wang et al., 2017). On the other hand, overexpression of the non-cellular autonomous phloem protein CmPP16 in pumpkin increases tolerance to drought, although the mechanism is not yet understood (Ramírez-Ortega et al., 2014).

A wealth of evidence now supports a model in which phloem-mobile RNAs act as critical components of gene regulatory networks involved in plant growth, defense, and crop yield at the whole plant level (Ham and Lucas, 2017). The supracellular protein CmPP16 was originally described as a phloem-mobile protein with non-specific RNA translocation capacity, which could also be transported long distance in heterografted cucumber on pumpkin rootstocks (Xoconostle-Cázares et al., 1999). This protein can dilate plasmodesmata facilitating the systemic movement of other molecules, so it is a key protein for engineering the translocation of macromolecules in the vascular stream to reach distant tissues.

Plants are continually challenged by pests and pathogens which can infect and colonize diverse tissues (Yadeta and Tomma, 2013). The vascular tissue is especially important since viruses and other pathogens (mostly bacteria and phytoplasma) use it as a conduit for their spread within the plants. Once entering the vasculature, microbial pathogens spread rapidly since they sequester photoassimilates, which in turn causes epinasty, chlorosis, and vascular wilting (Janse and Obradovic, 2010; Harwood et al., 2011). In response to biotic and abiotic stress, plants synthesize antimicrobial peptides (AMPs); heat shock cognate 71 kDa protein (HSC70; Pang et al., 2000); heat shock protein 90 (HSP90; Manaa et al., 2011); LEA dehydrins (Benešová et al., 2012; Kosová et al., 2013); pathogenesis-related proteins (Chakraborty et al., 2016); elements of the proteasome responsible for protein recycling (Lin et al., 2009); reactive oxygen species scavenging enzymes (Mostek et al., 2015); detoxification enzymes (Vítámvás et al., 2015); and defensins (Guerra-Lupián et al., 2018). Some of these proteins have been found in phloem exudates of different species, such as pumpkin (*Cucurbita maxima*; Lin et al., 2009), castor bean (*Ricinus communis*; Balachandran et al., 1997), rape (*Brassica napus*; Ostendorp et al., 2017), cucumber (*C. sativus*; Hu et al., 2016; Lopez-Cobollo et al., 2016), rice (*Oryza sativa*; Aki et al., 2008), melon (*Cucumis melo*; Malter and Wolf, 2011), and Arabidopsis (Batailler et al., 2012), as well as proteins involved in sieve tube maintenance.

Citrus species comprise perennial woody plants susceptible to both abiotic and biotic factors, including viruses, bacteria, fungi, oomycetes, and nematodes (Dalio et al., 2017). Among the most challenging diseases, Citrus greening or Huanglongbing (HLB) constitutes a serious threat to citriculture, although some varieties of Citrus species are asymptomatic to Citrus greening (Miles et al., 2017). In addition, several germplasms belonging to *Microcitrus warburgiana*, *Microcitrus papuana*, and *Microcitrus australis*, spp. *Eremocitrus glauca* and their hybrids, have been recently identified as potential sources of resistance against HLB (Alves et al., 2021).

Three *Candidatus* Liberibacter species have been identified as causative agents of HLB: *Candidatus* Liberibacter asiaticus (CLas; Bové, 2006), *Candidatus* Liberibacter americanus (CLam; Teixeira et al., 2005), and *Candidatus* Liberibacter africanus (CLaf; Jagoueix et al., 1994); being CLas the most aggressive and prevalent. CLas is an *in vitro* non-cultivable bacterium, which resides in the functional SE where the movement of the photoassimilates occurs. Therefore, the phloem sap translocation is blocked by the deposition of callose, a host defense response to the bacteria; as a result, starch accumulation in leaves is observed as diffuse mottle (Bové, 2006).

As mentioned earlier, AMPs are an important mechanism of innate immunity in plants (Ganz, 2003). Among those AMPs, plant defensins are small basic cysteine-rich peptides present throughout the plant kingdom that target mostly pathogen membranes. Secretion of defensins in root tips in *Heliophila coronopifolia* likely provides protection to root meristems against the attack of bacterial and fungal pathogens (Weiller et al., 2017). This suggests apoplastic rather than symplasmic translocation of defensins. Furthermore, defensins do not seem to be detectable in phloem sap exudates but present in diverse tissues (Rodríguez-Celma et al., 2016; Guan et al., 2020). This supports the notion that defensins are transported within tissues through classic secretion routes and would thus not coincide with the phloem translocation stream, where CLas resides.

Engineering protein translocation into the phloem stream by means of fusion to SE-resident proteins, such as CmPP16 (Guerra-Lupián et al., 2018) or its *Citrus* spp. ortholog, termed CsPP16, would allow the evaluation of different proteins in long-distance signaling. In particular, the capacity of providing supracellular capacities to AMPs would in principle protect plants against pathogens that reside in the phloem. The aim of this study was to identify and characterize *in silico* CsPP16 potential interaction with other proteins and to evaluate *in planta* its capacity to provide phloem-mobile properties to AMPs. CmPP16 ortholog in *Citrus sinensis* was identified and characterized. Its capacity to translocate AMPs to the vasculature conserving their antimicrobial activity was demonstrated. These plants were in turn analyzed for their capacity to mitigate CLas infection.

## Materials and Methods

### Plant Materials

Citrus plants were grown in a biosafety greenhouse under natural daylight conditions [summer: midday photosynthetically active radiation (PAR) of 1,500 μmol m^−2^ s^−1^, 42/20°C day/night temperatures, 16 h day length; winter: midday PAR of 1,000 μmol m^−2^ min^−1^, 32/20°C day/night temperatures, 12 h day length]. Plants were irrigated every 3 days and nutrients added in the irrigation water.

### Grafting Citrus Plants

In commercial citriculture, *Citrus tristeza virus* (CTV)-resistant rootstocks *Citrus volkameriana* and *Citrus macrophylla* are used. Mexican lime buds [*Citrus aurantifolia* (Christm.) Swingle] or sweet orange cv. “Valencia” [*C. sinensis* (L.) Osbeck] obtained from HLB-free plants were laterally grafted on the rootstock stem, at 60 cm above the pot, and a plastic bandage was located around the scion to protect the bud for 30 days. Grafted meristems were ringed along the stem of the rootstock for 1 month, until the scion became apical dominant. The stem above the scion was removed and plants were allowed to grow under the described conditions.

### Collection of Enriched Phloem Sap Exudate and Detection of Ortholog CsPP16

“Valencia” sweet orange plants grafted into *C. volkameriana* were maintained under greenhouse conditions at 25–30°C. Phloem sap extraction was carried out using the centrifugation technique described by Hijaz and Killiny (2014). To determine the transcript levels of different vascular-associated genes, phloem and foliar tissue obtained from plants of approximately 1-year-old were used. We also characterize the transcription levels of *CsPP16* in the following tissues: phloem sap, roots, juice, endocarp, stem, exocarp, leaf, flowers, and seeds, employing 3-year-old plants or fruits obtained from them. 30 μl of phloem sap, 300 μl of freshly extracted juice, or 100 mg of fresh tissue were used for RNA extraction using Direct-zol™ RNA MiniPrep (Zymo Research, Irvine, CA, United States). Real-time quantitative PCR (RT-qPCR) was performed with KAPA SYBR FAST One-Step Universal Kit (Roche, Baseloz, Switzerland) according to the manufacturer’s instructions using 100 ng of total RNA in 10 μl of final volume employing a StepOnePlus^™^ Real-Time PCR System (Life Technologies, Carlsbad, CA, United States). Glyceraldehyde-3-phosphate dehydrogenase (*CsGAPDH*) was used as reference gene, ribulose-bisphosphate carboxylase small subunit (*CsRbcS*) as negative control and phloem protein-2 (*CsPP2*), NAC domain-containing protein 87 (*CsNAC87*), and translationally controlled tumor protein-1 (*CsTCTP-1*) as controls of phloem expression in Citrus plants. All the primers used in RT-qPCR are listed in Supplementary Table 1. Relative expression was calculated by the (2^−ΔΔCt^) method (Livak and Schmittgen, 2001).

### Determination of CsPP16 *in silico* Interactor Proteins

The ortholog sequence of CmPP16 in *C. sinensis* (CsPP16) was identified through BLASTp using *C. maxima* protein PP16-1 (CmPP16, UniProtKB: Q9ZT47) as query. We also carried out the identification of Citrus orthologs for TCTP (GenBank accession no. XP_006489069.1), eIF5A (GenBank accession no. XP_006477598.1), RBP50 (GenBank accession no. XP_006473079.1), and HSC70 (GenBank accession no. XP_006486087.1). Subsequently, the prediction of the tertiary structure was carried out using trRosetta (Yang et al., 2020) and refined with Galaxy Refine Server (Giardine et al., 2005). The models were validated with PROCHECK v.3.5 (Laskowski et al., 1993) and ProSA (Wiederstein and Sippl, 2007) and visualized with UCSF Chimera X (Goddard et al., 2018). Protein–protein docking analysis was performed with HADDOCK 2.2 (van Zundert et al., 2015) to assess the possible interactions of CsPP16 with CsTCTP, CseIF5A, CsRBP50, and CsHSC70. We obtained 10 different poses for each molecular docking and the best pose was selected based on the lower energy score (Z-Score) and the RMSD parameter of each model.

### Plasmid Constructs

pB7FWG2 binary vector was modified to contain an expression cassette harboring the DNA sequences for human β-defensin 4 A precursor (GenBank accession no. NP_004933.1), the processed form of human lysozyme C chain A (GenBank accession no. AAA59535.1), *Xenopus laevis* magainin (GenBank accession no. NP_001081306.1), or *Drosophila melanogaster* cecropin (GenBank accession no. NP_524588.1) with Citrus-optimized codon usage and translationally fused to the Citrus CsPP16 (GenBank accession no. AAD05496.1) open reading frame (ORF). The expression of this gene fusion is driven by the *CaMV35S* promoter (p35S) and the *NOS* terminator (T-NOS). The complete expression unit was flanked by the left (LB) and right border (RB) from *Agrobacterium tumefaciens* T-DNA. The codon-optimized synthetic constructs were assembled by Genscript Corp. (Piscataway, NJ, United States) and cloned into the pUC57 vector, which harbors the ampicillin resistance gene.

### Experimental Design

Citrus plants were employed for transformation with the constructs coding for CsPP16-linker fused to the following AMPs: lysozyme, β-defensin, cecropin, magainin, β-defensin-GFP, and magainin-GFP to be employed as a reporter gene. In addition, 15 healthy isogenic plants of *C. aurantifolia* or *C. sinensis* grafted into *C. volkameriana* were also employed.

### Plant Genetic Transformation

Branches of healthy plants were selected and leaves and spines from the central branch were excised employing clean scissors. Agrobacterium cultures harboring the expression vector with the fusion proteins were placed into sterile cotton on the dormant buds and then wrapped with plastic. Fully irrigated plants were maintained into a plastic bag and the entire plant was covered with a large clear plastic bag for 48 h to maintain high humidity and favor the genetic transformation and emergence of newly transformed tissue. After 7 days, bandage was carefully removed, and branches were numbered using aluminum foil tags. After 3 months, emerging new leaves were evaluated.

### Transgene Detection by qPCR

Genomic DNA was obtained from 100 mg of leaf tissue samples collected from control and agro-infiltrated Citrus plants using the DNeasy extraction Plant Mini Kit (Qiagen). Total DNA was used as template to determine the transgene presence and to evaluate transgene/endogen ratio using real-time PCR (qPCR). Corresponding primers and probes for *CaMV35S* (p35Sf/p35Sr and p35Sp) and the cytochrome c oxidase (COXf/COXr and COXp) endogenous gene were employed (Supplementary Table 2). qPCR reactions contained 1× PCR buffer, 1.5 mm MgCl_2_, 250 μm dNTPs, 300 nm target primers, 180 nm target probe, 1× ROX Reference Dye (Thermo Fisher Scientific), 100 ng DNA, and 1 unit Platinum^®^ Taq DNA polymerase (Invitrogen, Carlsbad, CA, United States). Reactions were set on a StepOnePlus^™^ Real-Time PCR System (Applied Biosystems, Thermo Fisher Scientific Inc., Waltham, MA, United States) following the manufacturer’s instructions. The cycle conditions used were as follows: 94°C for 3 min followed by 40 cycles at 94°C for 15 s and 60°C for 60 s.

### RNA Quantification by RT-qPCR

Leaf RNA from young shoots from control and transformed plants was obtained as follows. The petiole was first removed, and the leaf blade was ground with a TissueLyser apparatus. Total RNA was purified with the RNeasy Mini Kit (Qiagen) according to the manufacturer’s instructions. Genetic material was incubated with DNase I (Promega, Madison, WI, Unites States) to remove contaminating DNA for real-time quantitative reverse transcriptase PCR (RT-qPCR) analysis. RT-qPCR was performed with a StepOnePlus^™^ Real-Time PCR System (Applied Biosystems^™^, Thermo Fisher Scientific Inc., Waltham, MA, United States). Briefly, DNase I-treated RNA was added to a 20 μl reaction mixture containing 10 μl of Kapa SYBR FAST qPCR master mix (Sigma-Aldrich), 10 μm primers, 200 ng of total RNA, and 1 μl ROX high (Bio-Rad, Hercules, CA, United States). Assay mixture was prepared with the primers enlisted in Supplementary Table 1. RT-qPCR conditions were as follows: 50°C for 30 min, 94°C for 2 min, and 40 cycles of 94°C for 15 s and 58°C for 35 s. Normalized mRNA accumulation was calculated employing the 2^−ΔΔCt^ method (Livak and Schmittgen, 2001) and obtained with the StepOnePlus software (Applied Biosystems).

### Confocal Microscopy

Leaves from Citrus plants transformed with the CsPP16-β-defensin-GFP or CsPP16-magainin-GFP fusion construct were collected, sectioned manually employing a surgical blade on a glass slide, to obtain the main vein and transversally sectioned in slices of 1 mm. Sections were submerged in water and analyzed immediately with a Leica confocal laser-scanning microscope (model TC-SP5/MO-TANDEM) at the LanSE facility in CINVESTAV-Mexico City, using a krypton/argon laser and the following filter settings: 488 nm excitation and 525 nm emission for green fluorescence, and 580/665 nm for chlorophyll autofluorescence. All images were recorded and analyzed with Leica Las AF software, followed by processing with Photoshop 8.0 software (Adobe), as described previously (Xoconostle-Cázares et al., 1999).

### Immunohistochemical Analysis

Immunohistochemical assays were performed as previously reported (Ruiz-Medrano et al., 1999) with the following modifications: fresh leaf tissue, sampled 60 days after local transformation with *A. tumefaciens* were immersed in fixative solution (FAA: 3.7% formaldehyde, 5% acetic acid, and 50% ethanol) for 12 h and dehydrated in ethanol series (30%, 50%, 70%, and 90%). Tissue was immersed overnight in 95% ethanol with 0.01% Safranin O. Two changes with 100% ethanol followed by two changes with acetone for 30 min each were performed, followed by an overnight incubation with 100% CitriSolv, and then gradual replacement with melted paraffin. 5 μm semi-thin cross-sections were obtained using a rotary microtome HM 315. Rehydrated sections were blocked with PBS, containing 1% BSA and incubated overnight with a 1:1,000 dilution of anti-human β-defensin polyclonal antibody produced in rabbit (200 μg/ml; Santa Cruz Biotechnology, Dallas, TX, Unites States). Sections were incubated in a secondary antibody (anti-rabbit IgG coupled to alkaline phosphatase -AP-, Santa Cruz Biotechnology) and rinsed under stringent conditions with PBS-Tween 20. The tissue was treated with NBT/BCIP until a blue signal was observed and the enzymatic reaction was then stopped by addition of 50 mm EDTA at pH 8.0. Sections were photographed with a DS-Ri1 camera (Nikon) mounted on an Optiphot-2 microscope (Nikon) and processed using NIS software (Nikon).

### Immunodetection of **β**-Defensin and Lysozyme

Western Blot assays were carried out following the protocol described by Hinojosa-Moya et al. (2013). Total protein extraction was performed from the main leaf vein of transformed plants using 500 mg of fresh tissue. Material was homogenized using the TissueLyser LT (QIAGEN) with 50 oscillations per second for 2–3 min. Tissue was suspended in 200 μl of protein extraction buffer, homogenized in vortex for 1 min, and centrifuged (14,000 rpm for 15 min at 4°C), the supernatant was placed in a new microtube (1.5 ml), and protein concentration was determined spectrophotometrically (Thermo Scientific NanoDrop 1000™) and adjusted to 20 μg/μl. Samples containing 200 μg of total protein were mixed with 2× denaturing buffer, heat treated at 90°C for 3 min, and resolved in 15% SDS–PAGE. Proteins in the acrylamide gel were transferred to PVDF membranes (Whatman^™^), previously activated with methanol (1 min), at 30 V for 5 h in transfer buffer, using the transfer system (Bio-Rad). Subsequently, the membrane was removed and placed in blocking solution 1× PBS, 5% skim milk, 0.1% Tween-20, followed by three washes of 5 min with 1× PBS and 0.1% tween-20. Then, the membrane was incubated overnight at 4°C in a solution containing the polyclonal antibody against lysozyme or β-defensin (Santa Cruz Biotechnology, CA, United States) diluted 1 in 1,000 of 1× PBS with Tween-20-0.1%. The next day, the first antibody solution was removed, and the membranes were washed three times for 30 min with washing solution (1× PBS, 0.1% Tween-20) to then be incubated with the secondary antibody IgG coupled to HRP (Santa Cruz Biotechnology) with a 1:2,000 dilution in PBS 1×-1% Tween 20 overnight at 4°C. The next day, the solution containing the second antibody was removed and three washes of 10 min each were performed with 1× PBS-0.1% Tween 20. Finally, the development of the membranes was performed by treating the membranes with Amersham ECL Western Blotting Detection Reagent (GE), following the manufacturer’s instructions.

### Quantification of CLas in Plant Samples

Total DNA was obtained using DNeasy kit (Qiagen) using 100 mg of leaf midvein. Bacterial viability was quantified by Ethidium MonoAzide-Treated DNA, followed by PCR (EMA-PCR), essentially as previously described (Trivedi et al., 2009). EMA enters to non-permeable dead cells and binds to DNA; it also binds to free DNA from dead, membrane-permeable bacteria. In the presence of light, EMA covalently binds to DNA, which cannot be longer used by Taq polymerase as template. Quantification of CLas from EMA-PCR represents live bacteria. A parallel PCR assayed with DNA without EMA treatment detects total bacteria. The arithmetic subtraction of total PCR minus EMA-PCR represents dead bacteria. Considering that EMA is light sensitive, aliquots were stored in dark microtubes, and DNA treatment was always performed in the dark, while its inactivation was also carefully assayed checking light intensity. In this case, two samples were assayed by triplicate, one corresponding to the direct DNA extraction and the other to EMA-DNA extraction, to calculate the ratio of dead and live cells to the total number of cells. To determine the number of CLas, a calibration curve was established using, as standard, a 700 bp 16S rRNA fragment from this bacterium. To this end, the recombinant-plasmid concentration was measured using a NanoDrop 2000c spectrophotometer (Thermo Fisher) and standardized from 2 × 10^7^ to 200 copies per PCR reaction. Plasmid copy number was calculated based on the molecular weight using the following formula: Number of copies = (Amount in ng × Avogadro’s Number)/(Length in bp × 1 × 109 × 650), considering an average molecular weight of 650 Da for each nucleotide. Real-time PCR reactions contained 240 nm target primer HLBas/HLBr, 120 nm target probe HLBp, 240 nm target primer internal control primer COXf/COXr, 120 nm target internal control probe COXp (Supplementary Table 2), 1× PCR buffer, 6.0 mm MgCl_2_, 240 μm dNTPs, and 1 unit Platinum^®^ Taq DNA polymerase (Invitrogen, Carlsbad, CA, United States). For qPCR, a StepOnePlus^™^ Real-Time PCR System (Applied Biosystems, Thermo Fisher Scientific Inc., Waltham, MA, United States) was used; conditions for this amplification were 94°C for 20 s followed by 40 cycles at 95°C for 1 s and 58°C for 40 s.

### Statistical Analysis

Data were analyzed with the GraphPad Prism statistical software (8.4.3 version). Graphic results are expressed as mean of triplicates. Bars represent standard deviation (SD). Significant statistical differences in RT-qPCR assays, comparisons between the different genes or tissues respect to leaf tissue were calculated using Student’s *t*-tests. A value of *p* less than 0.001 was considered statistically significant.

## Results

### The Pattern of Vascular Expression of *CsPP16* Is Comparable to That of Its Ortholog in Cucurbits

**Figure 1:**
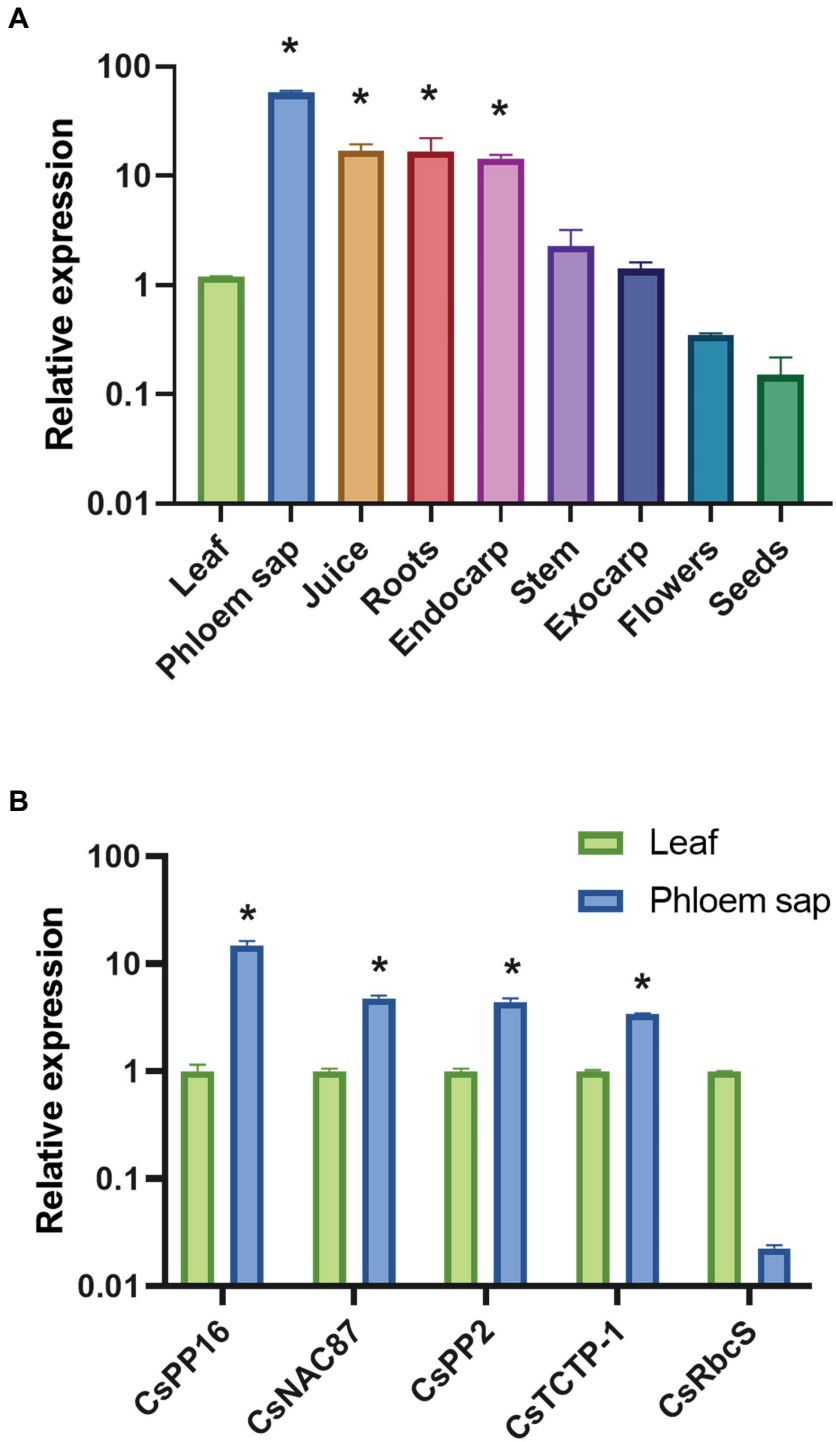
Relative accumulation of *CsPP16* mRNA in *Citrus sinensis* analyzed with Real-time quantitative PCR (RT-qPCR). Relative expression was normalized with the *GAPDH* transcript. **(A)** *CsPP16* accumulation profile in different tissues and floral organs of adult *C. sinensis* plants. **(B)** Comparison of *CsPP16* levels in young leaves and phloem of *C. sinensis* compared with other mRNAs potentially accumulating in the vasculature. Comparisons between the different genes or tissues with respect to leaf tissue were calculated using Student’s *t*-test (^*^p < 0.001).

To evaluate whether the *CsPP16* gene is the functional ortholog the cucurbit PP16, the accumulation of its mRNA in different tissues was evaluated. RT-qPCR was performed and normalized with the endogenous transcript *GAPDH* (*CsGAPDH*), which has been described as displaying a constitutive expression level (Mafra et al., 2012). The presence of *CsPP16* transcript levels were higher in phloem sap compared to other tissues. Likewise, the transcript was detectable in juice, roots, leaves, endocarp, stems, and tissues of the exocarp, while the lowest relative levels were observed in flowers and seeds (Figure 1A). This pattern is similar to *CmPP16* mRNA in pumpkin. Likewise, the relative expression of genes that have also been described as abundant in the vasculature of cucurbits was quantified. Young leaves and Citrus phloem were used in this assay. The quantification of the *CsNAC87*, *CsPP2*, and *CsTCTP-1* transcripts was consistent with previous reports of their detection in the vasculature (Figure 1B). In parallel assays for the characterization of mRNAs in vascular tissue, the *CsRbcS* mRNA has been used as a negative control to discard possible contamination from surrounding photosynthetic tissues. Considering its 63% similarity, the accumulation associated to the vasculature, and high expression levels in the analyzed tissues, CsPP16 is likely the functional ortholog of cucurbit CmPP16.

### *In silico* Analysis of Potential CsPP16 Interacting Proteins

**Figure 2:**
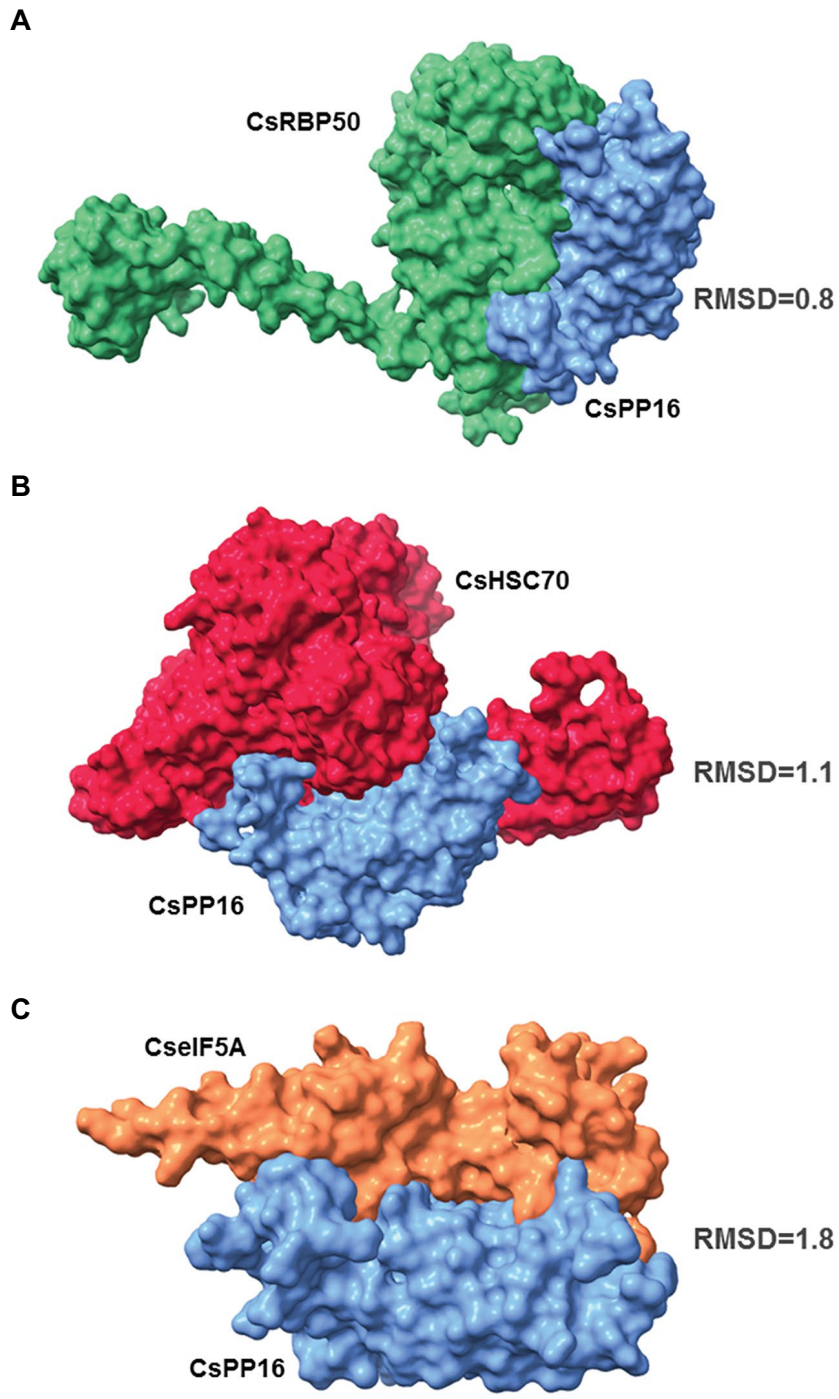
CsPP16 potentially interacts with phloem proteins as determined by molecular docking. Docking protein–protein graphs showing the interaction of CsPP16 (blue) with CsRBP50 (**A**, green), CsHCS70 (**B**, red), and CseIF5A (**C**, orange), *Citrus sinensis* orthologous of *Cucurbita maxima* proteins known to interact with CmPP16. Root Mean Square Deviation values are indicated next to each molecular docking model.

PP16 probably facilitates the transport of RNA and other proteins into the phloem translocation stream to exert their function in distant tissues. It has been shown previously that CmPP16 interacts with proteins and with RNA to form ribonucleoprotein complexes (Aoki et al., 2002; Ham et al., 2009). An *in silico* analysis was performed to analyze the potential interaction of CsPP16 with its corresponding orthologous proteins in Citrus RBP50, HSC70, TCTP, and eIF5A. The calculated simulations of the coupling suggested that CsPP16 interacts with CsRBP50 (Figure 2A), CsHSC70 (Figure 2B), and CseIF5A (Figure 2C). The molecular docking algorithms based on the conformation and the physicochemical complementarity at the protein–protein interface did not show a particularly strong interaction of CsPP16 with the CsTCTP (RMSD value: 14.7; Supplementary Table 3), although it cannot be ruled out that the *in vivo* interaction, if any, could occur mediated by an additional protein yet to be characterized.

### Design of Synthetic Genes to Mobilize Proteins to the Vasculature

To provide symplastic movement properties to proteins that are not present in the phloem, it was proposed to translationally fuse them to the CsPP16 protein. If its function is similar to CmPP16 (Guerra-Lupián et al., 2018), it could facilitate protein translocation to the vasculature. For this purpose, the *CsPP16* ORF was used, but removing the stop codon. Toward the CsPP16 3′ end, a linker sequence rich in glycine and alanine was inserted; this linker is a non-structured oligopeptide that facilitates the independent folding of the fused proteins (Wriggers et al., 2005). Peptides previously reported for having antimicrobial activity, encoded in different organisms, were selected as: β-defensin (human), magainin (frog), cecropin (insect), and lysozyme (human). The expression of these ORFs was directed by the *CaMV35S* promoter and *NOS* terminator. Citrus preferential codon usage was introduced in the constructs; therefore, the AMPs sequences were synthetic genes. Because the genetic transformation of Citrus was performed with *A. tumefaciens*, the left and right borders (LB and RB) were added to flank the complete expression unit. For the constructs expressing magainin and β-defensin, the GFP reporter protein was subcloned toward the 3′ end, also in translational fusion (Supplementary Figure 1).

### Identification of Citrus Transformants and Expression of the Mobile Transcripts and Proteins

Citrus buds from 1-year-old trees were previously grafted into rootstocks of *C. volkameriana* or *C. macrophylla*, both resistant to CTV. The lateral buds were genetically transformed as described (Guerra-Lupián et al., 2018) with the constructs encoding the CsPP16-AMP fusion proteins. Three months after transformation, the developed plantlets were analyzed by quantitative real-time PCR, amplifying the *CaMV35S* promoter and using the endogenous *COX* gene as internal control. Since transformed plants cannot be positively selected with a dominant selection marker, such as resistance to an antibiotic or herbicide, the transforming plants that contained copies of the *CaMV35S* promoter in a proportion equal to or greater than the copies of the endogenous *COX* gene were selected for further analysis. Of note, some plants are chimeras containing non-transformed tissues, which would result in *CaMV35S/COX* ratios lower than 1. Buds with copy number values *CaMV35S/COX* lower to 1 were discarded for use in the present work.

Table 1 shows the transformed plant lines analyzed according to transgene/endogen ratio. In general, *COX* amplification Ct values for Mexican lime samples ranged from 19.43 to 21.16, while for sweet orange from 18.01 to 19.87. For Mexican lime plants, *CaMV35S/COX* ratios extended from 1.1096 to 1.3159 for β-defensin transformed plants, from 1.2529 to 1.3399 for lysozyme, 1.222 for cecropin, and from 1.2556 to 1.2847 for magainin transformed plants. For sweet orange samples, *CaMV35S/COX* ratios ranged from 1.2043 to 1.3272 for β-defensin, 1.2104–1.2448 for lysozyme, 1.2406–1.4147 for cecropin, and 1.1231–1.2652 for magainin transformed plants.

**Table 1:** Positive *Citrus* transformed plants with the CsPP16-AMP fused proteins.

Citrus species	AMP^*^	No. of lines^**^
Mexican lime *Citrus aurantifolia* (Christm.) Swingle	β-Defensin	9
	Lysozyme	7
	Cecropin	1
	Magainin	4
“Valencia” sweet orange *Citrus sinensis* (L.) Osbeck	Defensin	6
	Lysozyme	2
	Cecropin	9
	Magainin	3

* AMP, antimicrobial peptide.

** Positive transformed plants analyzed by qPCR.

**Figure 3:**
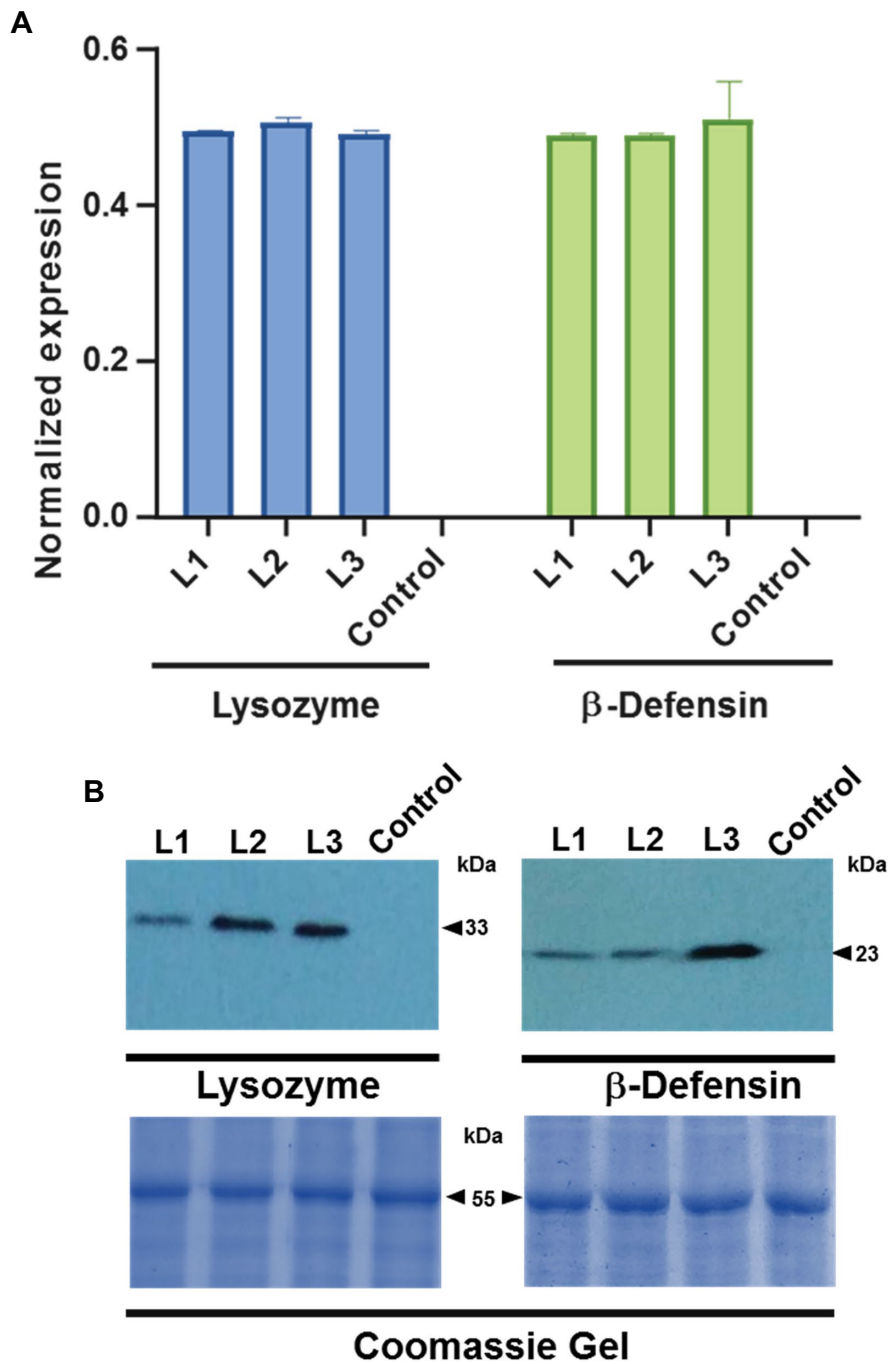
Detection of transcripts and fused proteins in Citrus plantlets. Three independent lines (L1-3) of plants transformed with lysozyme or β-defensin were analyzed. **(A)** RT-qPCR detection of CsPP16-lysozyme (left graph), and equivalent assay in CsPP16-β-defensin expressing plants (right graph). Relative expression was normalized with the *COX* transcript. Control, wild type Citrus was employed in both analyses. **(B)** Western blot analysis to detect the fused proteins in three independent transformants for lysozyme (left image) with a MW of 33 kDa and β-defensin (right image) with a MW of 23 kDa. Coomassie-brilliant-blue-stained SDS-PAGE gels indicating Rubisco Large Subunit (≈55 kDa) are shown as loading controls.

To demonstrate that the chimeric constructs were expressed, the accumulation of the mRNA of three independent lines of transformed plants with lysozyme and three transformed plants with β-defensin was measured by RT-qPCR. Figure 3A shows the presence of the *CsPP16-lysozyme* and *CsPP16-β-defensin* transcripts in the analyzed transformed plants. Detection values were normalized to the detection of the endogenous *COX* transcript and compared to a non-transformed control plant. To demonstrate that the transcript was efficiently translated, detection of the fusion protein was performed using commercial monoclonal antibodies directed against the human lysozyme or β-defensin proteins (Figure 3B). The fusion of CsPP16 with the linker and lysozyme had a molecular weight (MW) of 33 kDa, while the fusion protein with β-defensin had a MW of 23 kDa. Both proteins were detected when 200 μg of soluble protein was loaded, as described previously.

### CsPP16-Amps Are Mobile Proteins Present in the Citrus Vasculature

**Figure 4:**
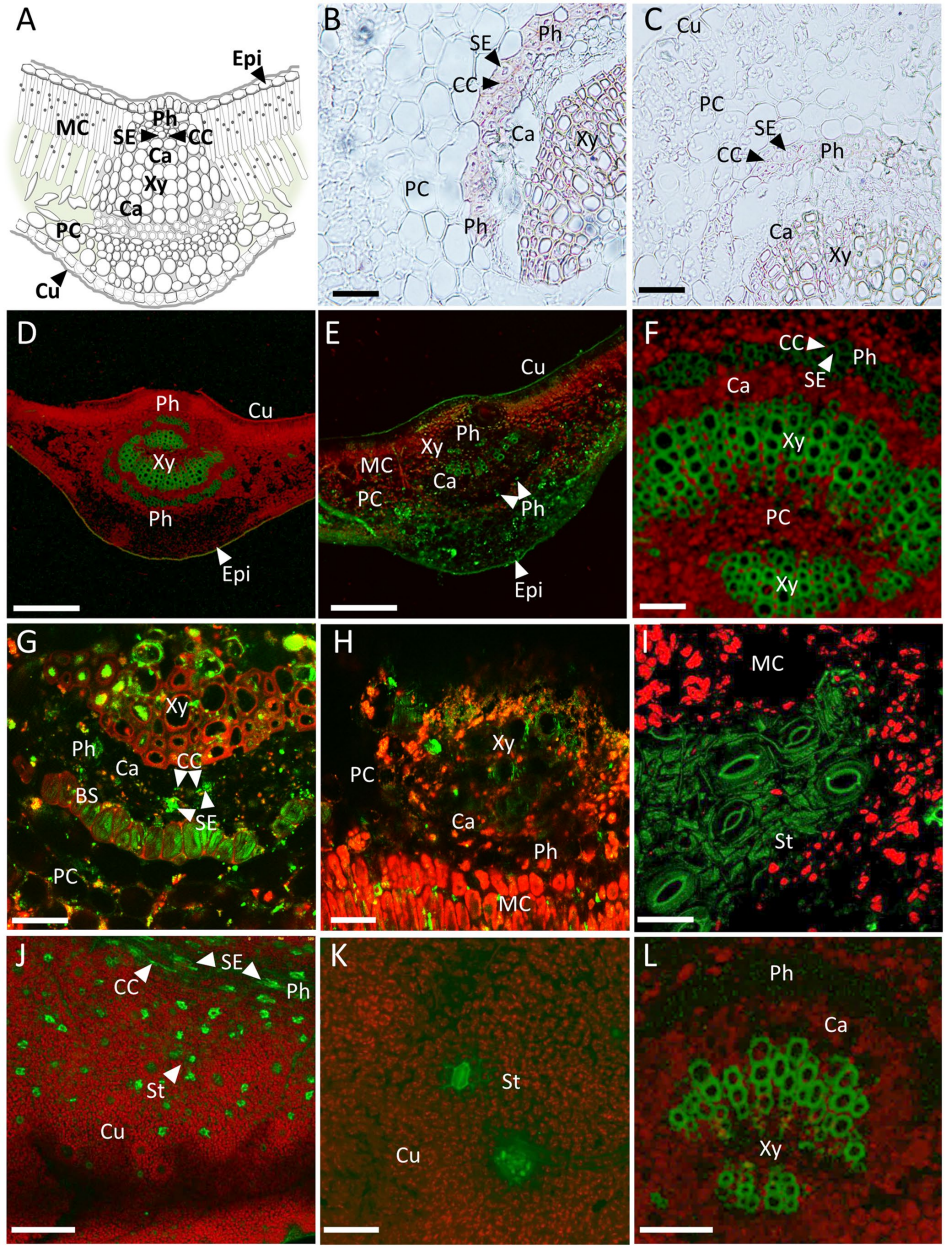
*In situ* localization of mobile CsPP16 translationally fused to antimicrobial proteins and GFP. **(A)** Schematic representation of transverse section of a central vein of Citrus leaf; **(B)** immunolocalization of β-defensin fused to CsPP16 in a leaf transverse section; **(C)** negative control of immunolocalization; **(D–F)** confocal images of transverse sections of leaves vein expressing β-defensin fused to CsPP16 and GFP; **(G,H)** transverse sections of main vein expressing CsPP16-magainin-GFP; **(I)** cuticle expressing same construct as in **(G,H)** images showing open stomata; **(J)** isolated mesophyll cells containing mobile proteins magainin fused to GFP, upper region showing a longitudinal fluorescent minor vein; **(K)** closed stoma on leaf surface containing the fused, mobile magainin; and **(L)** negative control displaying autofluorescence of xylem tissue. BS, Bundle Sheath; Ca, Cambium; CC, Companion Cell; Cu, Cuticle; Epi, Epidermis; MC, Mesophyll Cell; MV, Minor Vein; Ph, Phloem; PC, Phloem Parenchyma; SE, Sieve Element; St, Stoma; and Xy, Xylem. Scale bar for panels **(B,C,F,I,L)** = 50 μm; **(D,E,J)** = 250 μm; **(G,H)** = 25 μm; and **(K)** = 100 μm.

To evaluate the presence of CsPP16 in vascular tissue, particularly in phloem, indicating its capacity to translocate functional cellular proteins to the vasculature, two approaches were used as: *in situ* immunolocalization assays of the CsPP16-β-defensin fusion, using commercial anti-β-defensin antibodies, and confocal analysis of β-defensin or magainin fused to GFP. Figure 4A shows the schematic representation of transverse section of a central vein of Citrus leaf. Figure 4B shows the immunolocalization of β-defensin fused to CsPP16 in a transverse section displaying β-defensin presence in the cells of the functional phloem, indicated with a dart. Both Companion Cell (CC) and Sieve Element (SE) are shown containing the purple signal associated to β-defensin detection. At the sensitivity levels of the technique, it was undetectable in the parenchymal or cambium cells. The accumulation in the CC and in the SE indicated its phloem transport. A cross-section of a non-transformed control is shown without the signal associated to vascular tissue (Figure 4C), suggesting that the signal observed in Figure 4B was specific. The signal observed in the xylem in both Figures 4B,C shows the non-specific absorption of the chromogenic reagent employed in this assay. The second technique used to analyze its expression in the Citrus vasculature was to obtain plants expressing the CsPP16- β-defensin (Figures 4D–F) or CsPP16-magainin (Figures 4G–K) proteins fused to GFP and to evaluate the fluorescence-associated *in situ* localization of the reporter protein. As evidence of their active translocation, the main vein of photosynthetic leaves of plants transformed with the fused proteins were analyzed by confocal microscopy. In Figure 4D, a cross-section of Citrus leaf expressing β-defensin fused to CsPP16 and GFP with green fluorescence located in the axial and abaxial phloem is observed, the xylem showed autofluorescence in the central region, as occurred with the control in Figure 4L. The presence of GFP was evident in Figure 4E where, in addition to its clear presence in the vascular area, there was also a signal associated with parenchymal cells and in the mesophyll cells of the leaf abaxial region. Phloem cells containing the fluorescent signal are indicated with darts. Figure 4F shows with higher resolution the vascular area of a plant expressing the mobile β-defensin fused to CsPP16 and GFP, where it is possible to observe the phloem tissue, including CC and SE with a green, fluorescent signal. On the other hand, Figure 4G shows a transverse section of main vein expressing magainin-CsPP16-GFP with signal in the vasculature, in which two SEs are shown. CCs are distinguished as thin long cells, in close boundary to SE, also containing fluorescent GFP-associated signal. In addition, there was signal in the Bundle Sheath (BS). Figure 4H shows signal in the vascular area, but also in mesophyll cells. Figure 4I shows the surface of a leaf near the central vein, observing a set of open stomata containing GFP, also with possibly auto-fluorescent cuticle; a weak signal could be observed in the cells of the mesophyll. Figure 4J shows minor veins with GFP-associated fluorescence, as well as a mid-order vein in longitudinal position (upper part of the image) where CC and SE were observed, both containing GFP. CCs in the transverse section are thin, obliques cells; while SEs are rounded, bigger, with a green, and high fluorescent signal. Figures 4I,K showed independent fluorescent cells, some of which can be identified as stomata, but others are possibly mesophilic cells actively expressing the CsPP16-magainin-GFP fusion. The control in Figure 4L shows the autofluorescence of the xylem, and the vascular area devoid of signal, as well as the parenchyma cells. The evidence presented here confirmed the property of CsPP16-β-defensin-GFP and CsPP16-magainin-GFP to translocate to long distance and accumulate in the vasculature of adult Citrus plants.

### Antimicrobial Peptides Were Active When Mobilized to the Vasculature in Citrus Trees

**Figure 5:**
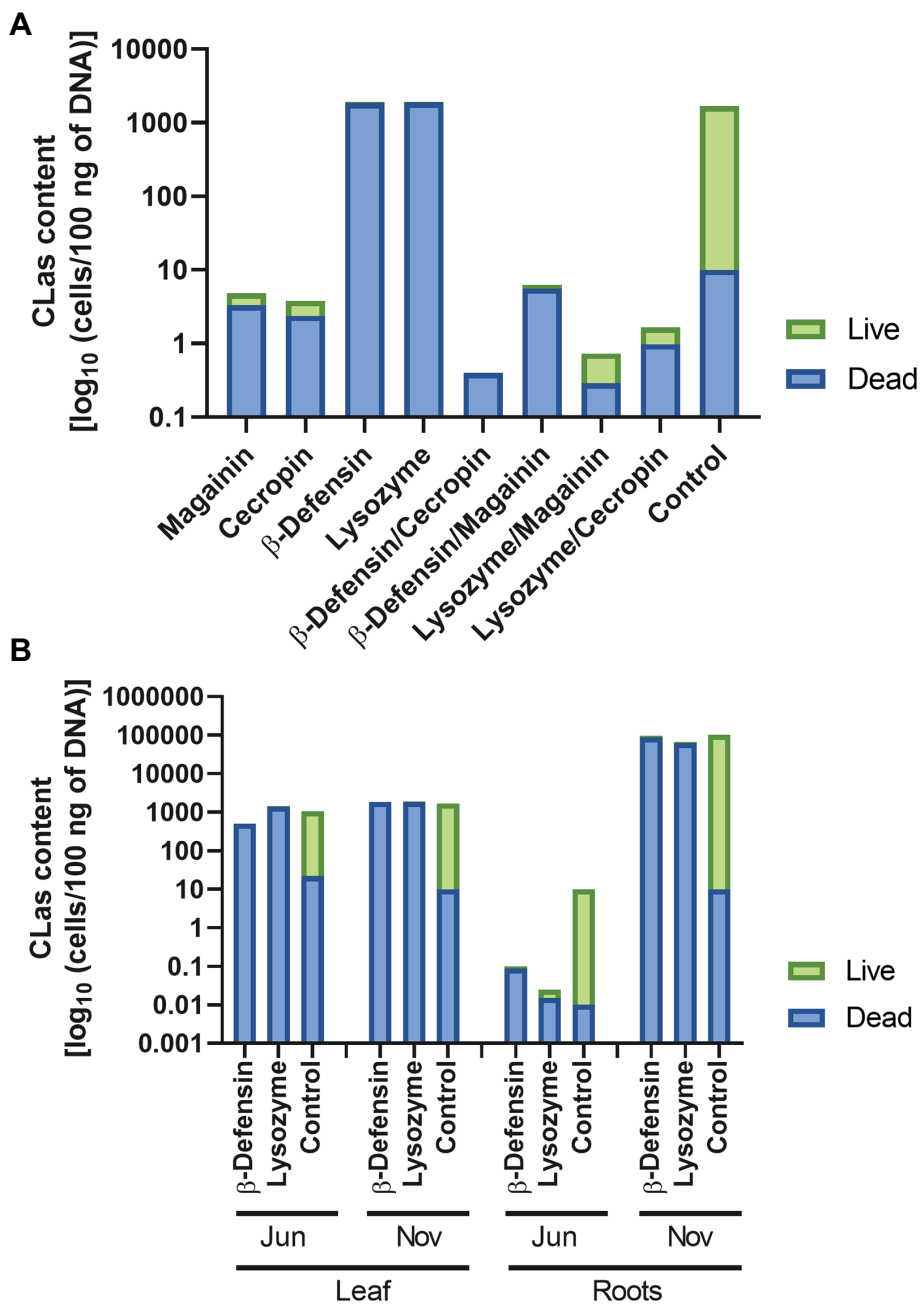
Activity of antimicrobial peptides translocated to the vasculature in HLB-infected Citrus trees. Quantitative Real-Time calculated CLa content as log_10_ (cells/100 ng of DNA). Live or dead bacteria were estimated using EMA-PCR **(A)** content of *Candidatus* Liberibacter asiaticus in Citrus trees expressing magainin, cecropin, β-defensin, lysozyme, and combinations: β-defensin/cecropin, β-defensin/magainin, lysozyme/magainin, and lysozyme/cecropin. Control plants are untransformed, wild type Citrus trees. **(B)** Kinetics of selected β-defensin and lysozyme expressing plants were assayed during a year, measuring CLa levels in June and November, in both leaves and roots. Complete bars represent total bacteria content, green bar is live bacteria, and blue bar is dead bacteria content.

The accumulation of CmPP16 and its Citrus ortholog in the sieve tube, as well as its ability to mediate protein and RNA long-distance transport, suggested the possibility of engineering proteins of interest to mobilize them into the vascular tissue for basic research as well as for biotechnological applications. Our interest in providing translocation capacities to proteins with antimicrobial activity was based on the necessity to control or mitigate diseases of the plant vasculature, especially the phloem. However, despite the evidence of their entry into the vasculature, the question about the functionality of mobile peptides was still unanswered. The translocation of proteins fused to CmPP16 has been reported previously (Taoka et al., 2007; Guerra-Lupián et al., 2018), and in the present work, we demonstrated that translational fusions toward the C-terminus of CsPP16 still allowed the long-distance translocation of GFP. It was thus of interest to evaluate the antimicrobial capacity of the fused proteins magainin, cecropin, lysozyme, β-defensin, and some combinations of them in Citrus trees infected with HLB. Citrus trees expressing these antimicrobials were challenged to populations of the insect vector *Diaphorina citri* infected with CLas, for a period of 1 year. The results showed that these plants had a significant reduction in live bacterial content when compared to the control group (Figure 5A). RNA, and possibly DNA, is present in phloem exudates (Xoconostle-Cázares et al., 1999), and the ribonucleoproteins complexes formed in the phloem sap protected these nucleic acids from degradation, substantially lengthening their half-life. If the presence of CLas bacterial DNA was detected, it would be important to know if it belonged to live cells or DNA from a lysed cell. Therefore, the amount of live and dead bacteria to discriminate among these two scenarios was evaluated. In Figure 5A, the proportion of dead bacteria was higher in trees expressing antimicrobials and their combinations, for example, trees expressing magainin or cecropin had the lowest CLas content when individual AMPs were assayed. Likewise, the bacteria detected in plants expressing β-defensin and lysozyme was similar to the control plants, but the major bacteria detected were dead. When antimicrobial combinations were evaluated, the lowest amounts of CLas could be observed, for example, in the plants expressing β-defensin/cecropin, β-defensin/magainin, lysozyme/magainin, and lysozyme/cecropin combinations. Considering possible biotechnological applications, antimicrobials with the capacity to mitigate the infection by CLas that are innocuous for humans were considered; namely, human β-defensin and lysozyme. These would not induce immune rejection in the case of human consumption. Thus, its capacity to control CLas was measured for another year in a biosafety greenhouse in Tecomán, Colima, Mexico, which is the largest area where Mexican lime is grown, as well as a region where HLB has become endemic. The quantification of CLas was carried out in the months of June and November 2018 (Figure 5B). The first corresponds to the beginning of summer, the time of highest temperature in this region, and the latter, when the temperature has decreased. During the warmest months, when temperatures of 45°C are reached regularly, a substantial decrease in CLa has been observed in plants producing antimicrobials, but also in controls, we had observed this phenomenon in the epidemiology of this bacterial disease in 2016 and 2017 (Lopez-Buenfil et al., 2017). In parallel tests, the number of bacteria present on the leaves and roots was quantified. The results obtained indicated the presence of mostly dead bacteria when compared to control trees. Despite this fluctuation in the amount of CLas in warm and temperate climates and in source tissues, such as leaves, and sink tissues, such as roots, the trees maintained their photosynthetic capacity and their production (data not shown). However, the diffuse mottling on the leaves, a characteristic symptom of the disease, remained in the plants.

## Discussion

The notion of the movement of informational macromolecules into the vascular tissue to coordinate differentiation processes and responses to biotic and abiotic stress has opened a new field in the study of plant developmental biology. Diverse molecules are transported through the phloem translocation stream, mostly photoassimilates but also hormones, and different secondary metabolites; however, certain informational macromolecules, such as proteins and nucleic acids, are also transported through the same pathway (Campbell and Turner, 2017). In angiosperms, in which the SEs are enucleated, the CCs synthesize these macromolecules which are loaded into the phloem translocation stream, some of which are required for SE maintenance and others reach distant organs to exert their function (Ruonala et al., 2017). Macromolecules are transported as protein and ribonucleoprotein complexes, which would then exit the phloem and enter cell types whereby they could regulate cellular and physiological functions in distant tissues. The classic example is represented by the florigen, first described in 1937 by Chaïlakhyan (1985) as a molecule synthesized in mature leaves and transported to the apex where it induced flowering. After decades of research, it is well established that florigen is a protein or a ribonucleoprotein complex, which is synthesized in source leaves, is graft-transmissible and reaches the shoot apical meristem *via* the phloem to induce flowering (Lin et al., 2007). Similar phenomena involving long-distance signaling *via* the phloem have been thoroughly described, such as tuberization, phosphate sensing, drought, and pathogen resistance; the signaling molecules associated are microRNAs, mRNAs, or proteins acting in coordinated networks (Corbesier et al., 2007; Pant et al., 2008; Navarro et al., 2011; Ramírez-Ortega et al., 2014; Wang et al., 2017; Yan et al., 2020). A first report of a supracellular protein with non-specific RNA translocation capacity suggested a mechanism for phloem transport of endogenous proteins and RNAs, mechanistically related to the systemic movement of both RNA and DNA viruses (Xoconostle-Cázares et al., 1999). Indeed, CmPP16 can facilitate the movement of RNA through plasmodesmata, is found in the sieve tubes, and can form *in vivo* and *in vitro* complexes with other proteins and RNAs conceivably with supracellular functions. The accumulation of the protein and its mRNA in the phloem sap suggests that its synthesis occurs in vascular cells, mostly in the CC, although *in situ* hybridization demonstrates the detection of its mRNA also in cambium cells, a non-differentiated meristematic tissue, which will later give rise to the specialized vascular tissues: phloem and xylem (Xoconostle-Cázares et al., 1999; Ruiz-Medrano et al., 2001).

The identification of proteins with similar functions in other species, in particular those of agronomic relevance, may allow the dissection of this selective transport system and redirect the movement of cell-autonomous proteins of interest to the vascular tissue and investigate its function. In the present work, Citrus species were selected as an experimental model to provide trafficking properties to antimicrobial proteins to reach the vasculature. For this purpose, the supracellular protein PP16 was selected as carrier, for which the gene encoding its Citrus ortholog CsPP16 was identified. This had 63% identity with CmPP16, as well as the characteristic C2 signature. The accumulation of its mRNA in the phloem sap was the highest among the tested tissues. The transcript was also detected in juice, roots, and endocarp, although in lower levels in stems and exocarp. The levels of this transcript in flowers and seeds were undetectable, similar to *CmPP16*. Additionally, other mRNAs likely present in Citrus phloem sap (*CsNAC87*, *CsPP2*, and *CsTCTP-1*) were detected, albeit to much lower levels than *CsPP16* mRNA. In contrast, *CsRbcS* mRNA showed the lowest levels, supporting the notion that the exudates were rich in phloem content. This evidence suggested that Citrus sap obtained with low centrifugal force was equivalent to cucurbit sap exudates. In addition to the accumulation in phloem sap, the possible interaction of CsPP16 with *C. sinensis* orthologs of *C. maxima* proteins known to interact with CmPP16 was evaluated *in silico*, since the formation of protein–protein or ribonucleoprotein complexes is required for their phloem transport (Ham et al., 2009). Molecular docking algorithms based on conformation and physicochemical complementarity at the protein–protein interface suggested an interaction between CsPP16 with HSC70, CsRBP50, and eIF5A. In contrast, when the interaction with CsTCTP was calculated, the values obtained did not suggest a high affinity interaction; these data were consistent with previous reports (Aoki et al., 2005).

It was proposed herein to generate translational fusions of proteins of interest with the carrier CsPP16. The design included the *CsPP16* ORF, driven by the constitutive *CaMV 35S* promoter. An unstructured linker rich in glycine and alanine between CsPP16 and the AMP fusions was added to allow their independent folding (Wriggers et al., 2005). The selection of peptide antimicrobials was based on their known ability to destabilize bacterial plasma membranes (human β-defensin, *X. laevis* magainin, and insect cecropin A), as well as to digest bacterial cell wall (human lysozyme; Scanlon et al., 2010; Kumar et al., 2018). Although the *CaMV35S* promoter is constitutive and generally non-tissue specific, proteins with cell-to-cell and long-distance movement capacity could in principle be transported to the phloem translocation stream. Since no genetic selection markers were used after transformation, an exhaustive screening was carried out to identify the transformed plants containing the vector of interest. The transformation of plants without a dominant selection marker could generate genetic mosaics even if an axillary meristem was transformed, so putatively transformed plantlets were screened using real-time PCR with an endogenous marker gene. Transformed plants with β-defensin and lysozyme were further evaluated for the presence of mRNAs, normalized with the endogenous *COX* transcript, showing that the constructs were efficiently transcribed. The antimicrobials magainin and cecropin were evaluated by conventional RT-PCR, showing that the constructs were also transcribed in these independent lines (data not shown). The evaluation of protein synthesis was demonstrated by Western blot assays, using protein extracts from the central vein of photosynthetic leaves of transformed plants. The detection of the size expected bands for CsPP16-β-defensin or CsPP16-lysozyme fusion proteins suggested an adequate translation of the genetic construct. In transformed plants with the CsPP16-AMP-GFP constructs, GFP-associated fluorescence was detected in the phloem area of the central veins analyzed. Likewise, it was observed in mesophyll, vascular bundle, and in phloem parenchyma. On a speculative note, the observed signal might correspond to protein in transit to the phloem. The presence of the signal associated with stomata was intriguing, since these specialized cells are symplastically isolated. It was possible that this prevented their plasmodesmata and/or symplasmic transport, resulting in its accumulation and thus higher fluorescence levels than in the surrounding cell types. Evidence of its localization in vascular tissue confirmed the hypothesis that the carrier protein CsPP16 might facilitate the transport of macromolecules to the vasculature, with their possible long-distance movement, likely from source to sink tissues in adult Citrus plants.

The most likely mechanism of β-defensin, magainin, and cecropin action is to disrupt membrane permeability. After interacting with bacterial cell membranes through electrostatic interactions, peptides bind to the membrane surfaces with their hydrophobic sides anchored in the hydrophobic lipid core of the bilayer. They can induce damage and unregulated permeability of larger molecules, such as proteins, eventually resulting in cell death (Lei et al., 2019). Antimicrobials and CsPP16 are hydrophilic in nature, which can help the former to bind the membrane surface and interact with the hydrophobic region of the bacterial membrane, resulting in its destabilization. On the other hand, the control of pathogenic bacteria with lysozyme under high hydrostatic pressure has been reported (Masschalck et al., 2001). This high-pressure condition is present in sieve tubes. It has also been shown that β-defensin which forms pores in the membrane of target organisms, mainly eubacteria and fungi, conferred resistance to different fungi when expressed in Arabidopsis (Aerts et al., 2007).

Although AMPs are widely distributed in most organisms, including plants, phloem sap exudate proteomes appear to lack these proteins. The possibility of mobilizing AMPs to phloem sap therefore involves modifying the plant innate immune response against phytopathogenic bacteria. However, having demonstrated its presence in vascular tissue, the open question was whether the proteins would have correctly folded and were biologically active. Infection of plants expressing phloem-mobile antimicrobials with HLB was possible by identifying an area of commercial orchards where this disease is endemic. In a biosecurity area in the region of Tecomán, Colima state, in Mexico, the psyllid insect *D. citri*, the vector that transmits CLas, was allowed to enter and infect plants. After 1 year of evaluation, the plants were found to be infected, but having a significant reduction in live bacteria content compared to the control group. The evaluation of dead and viable bacteria allowed us to interpret that a proportion of the copies detected by qPCR corresponded to DNA from dead bacteria, showing an association between the supracellular expression of antimicrobials and the death of CLas.

The evaluation of different antimicrobials confirmed their ability to control bacteria. However, in terms of perception of the potential use of an antimicrobial, the present study was followed with plants expressing two AMPs of human origin, the β-defensin and lysozyme. The rationale for this selection also considered that potential consumers would not be in contact with new potentially immunogenic proteins that would challenge their immune system. The measurement in two seasons of the year was to contrast the influence of the temperature in that tropical area, where at the beginning of summer temperatures can reach 45°C, where CLas propagation is limited, while at the end of autumn, the bacteria increase their population (Lopez-Buenfil et al., 2017). The antimicrobial-expressing trees maintained agronomic characteristics compatible with their health, when compared with the control trees which showed death of some branches and lower production (data not shown). Despite the significant decrease in bacteria in plants that expressed mobile antimicrobials, the presence of diffuse mottling on their leaves, a characteristic symptom of the disease, remained in the evaluated plants with differences in intensity depending on the time of year. This strategy is proposed to mitigate the disease associated to CLas presence; however, the plant hypersensitive response is still provoking accumulation of callose. CLas is not the only pathogen threatening Citrus crops, but also *Xanthomonas citri,* the causative agent of Citrus canker and which colonizes the xylem (Hao et al., 2016). It will be of interest to evaluate the capacity of AMP-producing plants in the control or mitigation of this microorganism. Regarding the undesirable decrease in beneficial bacterial population because of the effect of the expression of AMPs, it should be noted that the movement of these peptides should occur within the symplasic domain, and there would be no secretion by the classical routes of these molecules to be then present on the surface of the phylloplane or exudated by the root system. The assessment of the microbial diversity of bacteria and fungi in the root of Citrus expressing supracellular, symplastic antimicrobials has already been carried out (manuscript in preparation).

Plant breeding in the last century has employed genetic tools focusing on dominant disease and pest resistance traits in commercial genotypes (Francis et al., 2017). On the other hand, the strategy for increasing productivity has been in many cases to engineer carbohydrate partitioning in plants. This has been done by modification of apoplastic carbon allocation by overexpression of sugar transporters or the use of phytohormones and nucleotides (Hennion et al., 2019). The potential manipulation of carbon partitioning *via* the symplasm by facilitating the supracellular translocation of macromolecules could be engineered with carrier proteins, such as CsPP16. This protein family is present in all land plants; on a speculative note, parallel mechanisms for ribonucleoprotein phloem translocation could be present in other plant models and are thus also susceptible to engineering supracellular movement *via* the symplasm. The experimental model described in the present research opens the possibility of providing supracellular translocation properties to cell-autonomous proteins of interest, through fusions with CsPP16; this could allow to redesign of signaling routes in response to environmental cues *via* vascular mobilization of cellular proteins for basic research as well as for biotechnological applications.

## Data Availability Statement

The original contributions presented in the study are included in the article/Supplementary Material, further inquiries can be directed to the corresponding authors.

## Author Contributions

RR-M and BX-C: conceptualization and funding acquisition. BX-C, RR-M, BC-P, JR-P, LN-M, BV-H, and DJ-L: methodology. BX-C, RR-M, BC-P, JR-P, LN-M, BV-H, AC-R, and ML-V: software, validation, and formal analysis. BX-C, RR-M, BC-P, JR-P, LN-M, and BV-H: investigation. BX-C, RR-M, and BC-P: writing, review, editing, and supervision. BX-C: project administration. All authors have read and agreed to the published version of the manuscript.

## Funding

The research was supported by the Grant SENASICA 2016–2018 to BX-C and RR-M. JR-P (483659) and DJ-L (781282) are CONACYT Mexico fellows.

## Conflict of Interest

A patent file was registered in the Mexican Institute for Intellectual Protection.

The authors declare that the research was conducted in the absence of any commercial or financial relationships that could be construed as a potential conflict of interest.

## Publisher’s Note

All claims expressed in this article are solely those of the authors and do not necessarily represent those of their affiliated organizations, or those of the publisher, the editors and the reviewers. Any product that may be evaluated in this article, or claim that may be made by its manufacturer, is not guaranteed or endorsed by the publisher.
