# Supplementary material for: Engineering Macromolecular Trafficking Into the Citrus Vasculature
Source: Front Plant Sci. 2022 Feb 1;13:818046. doi: 10.3389/fpls.2022.818046 (PMC8844563; doi:10.3389/fpls.2022.818046)
Supplement: Supplementary file 1 [file Data_Sheet_1.docx]

Supplementary Material

***Supplementary Figures***


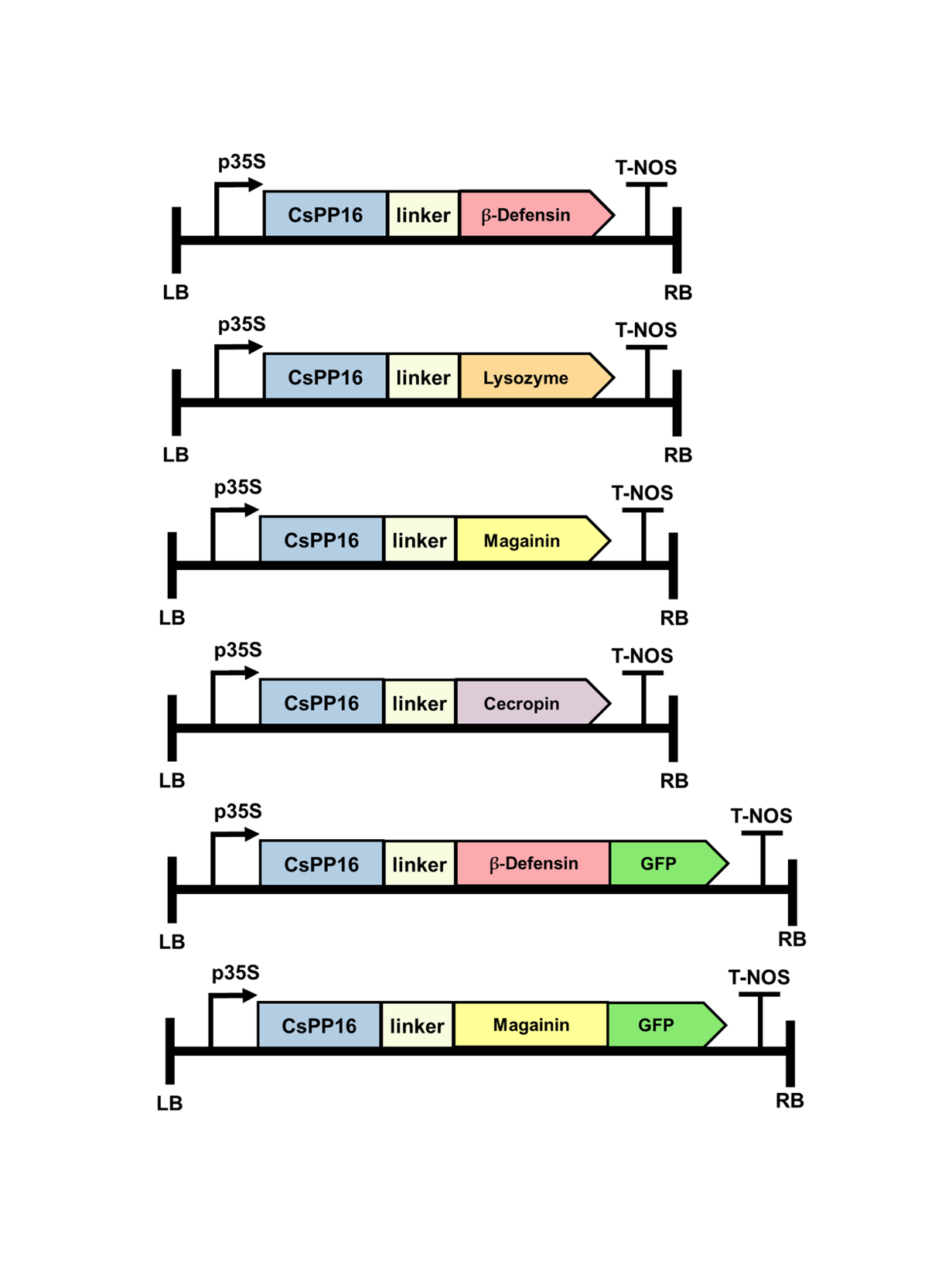


**Figure S1. Design of synthetic genes to mobilize proteins to the phloem.** The *Cauliflower Mosaic Virus* 35S promoter (p35S) directed the expression of the translational fusion of the CsPP16 protein with the antimicrobial peptides (AMPs) defensin, magainin, cecropin and lysozyme. An unstructured peptide that functioned as a linker was encoded between CsPP16 and the AMP. *Agrobacterium tumefaciens* *nopaline synthase* terminator (T-NOS) was the transcription terminator of these genes. The GFP reporter gene was translationally linked to the CsPP16-linker-magainin and defensin encoding gene. LB: Left border and RB: Right border of Ti plasmid of *A. tumefaciens.*

***Supplementary Tables***

**Table S1**. Primer sequences used in RT-qPCR experiments.

| **Name** | **Sequence (5’ to 3’)** | **Product (bp)** | **Reference** |
| --- | --- | --- | --- |
| CsRbCS F | TGCAACCCAGGTTTTGAAGGAG | 120 | This work |
| CsRbCS R | TTGGCAGCAAGGAAACTGATGC |  |  |
| CsTCTP-1 F | TGGTGCAAATCCTTCTGCCGAAG | 75 |  |
| CsTCTP-1 R | ATGTCAACCACCTTCACGGCTTG |  |  |
| CsNAC87 F | AATTTGGAGAGCAGCAGCCAGTG | 71 |  |
| CsNAC87 R | TGTGAGCGGCTGATCATGGATTG |  |  |
| CsPP16 F | TGGTGAAGCCACAATCTATG | 75 |  |
| CsPP16 R | CGAGAATGTAGTTCAGCAGTTC |  |  |
| CsGAPDH F | TCTTGCCTGCTTTGAATGGA | 80 | Carvalho et al., 2010 |
| CsGAPDH R | TGTGAGGTCAACCACTGCGACAT |  |  |
| CsPP2 F | TGCAGCATTTGGATGGGAAGCAC | 78 | Miyata et al., 2017 |
| CsPP2 R | ACTTGGCGATTCTGCACTTTCCC |  |  |
| Def F | TATGCCACTTCCAGGTGTTT | 80 | This work |
| Def R | AAACTGGATGGCAAATAGCAC |  |  |
| Lys F | CACCAGGTGCTGTTAATGCT | 146 |  |
| Lys R | ATTTCTCCAAGCAACCCAAG |  |  |
| Cox F | GTATGCCACGTCGCATTCCAGA | 68 | Li et al., 2006 |
| Cox R | GAATGCCCTTAGCAGTTTTGGC |  |  |

**Table S2**. Primer sequences used in transgene detection by qPCR.

| **Name** | **Sequence (5’ to 3’)** | **Product (bp)** | **Reference** |
| --- | --- | --- | --- |
| p35Sf | GCCTCTGCCGACAGTGGT | 82 | Waiblinger et al., 2008 |
| p35Sr | AAGACGTGGTTGGAACGTCTTC |  |  |
| p35Sp | FAM-CAAAGATGGACCCCCACCCACG-TAMRA |  |  |
| COXf | GTATGCCACGTCGCATTCCAGA | 68 | Li et al., 2006 |
| COXr | GCCAAAACTGCTAAGGGCATTC |  |  |
| COXp | TET-ATCCAGATGCTTACGCTGG-BHQ |  |  |
| HLBas | TCGAGCGCGTATGCAATACG | 75 |  |
| HLBr | GCGTTATCCCGTAGAAAAAGGTAG |  |  |
| HLBp | FAM-AGACGGGTGAGTAACGCG-TAMRA |  |  |

**Table S3:** Statistical analysis for docked complexes between CsPP16 and *C. sinensis* orthologs of *C. maxima* proteins known to interact with CmPP16

|  | **HADDOCK score (a.u.)** | **RMSD* (Å)** | **Van der Waals energy (E_vdw_) (kcal mol^-1^)** | **Electrostatic energy (E_elec_) (kcal mol^-1^)** | **Desolvation energy (E_desol_)**  **(kcal mol^-1^)** | **Z-score** |
| --- | --- | --- | --- | --- | --- | --- |
| CsTCTP | 156 | 14.7 | -84.2 | -470.3 | 10.4 | -1.6 |
| CseIF5A | 65.3 | 1.8 | -91.2 | -730.1 | 11.4 | -1.9 |
| CsRBP50 | 86.7 | 0.8 | -66.9 | -778.1 | 14.8 | -1.6 |
| CsHSC70 | 172.2 | 1.1 | -67.9 | -765.3 | 29.3 | -1.5 |

*RMSD= Root Mean Square Deviation

# References

Carvalho, K., de Campos, M., Pereira, L., and Vieira, L. (2010). Reference gene selection for real-time quantitative polymerase chain reaction normalization in ‘‘Swingle’’ citrumelo under drought stress. *Anal. Biochem.* 402, 197–9. doi: 10.1016/j.ab.2010.03.038

Li, W., Hartung, J., and Levy, L. (2006). Quantitative real-time PCR for detection and identification of *Candidatus* Liberibacter species associated with citrus huanglongbing. *J. Microbiol. Methods*. 66, 104e15. doi: 10.1016/j.mimet.2005.10.018

Miyata, L., Harakava, R., Attilio, L., Mendes, B., Januzzi, M., Lopes, J., et al. (2017). Phloem promoters in transgenic sweet orange are differentially triggered by *Candidatus Liberibacter asiaticus*. *Rev. Bras. Frutic.* 39, e-993. doi: 10.1590/0100-29452017 993

Waiblinger, H., Ernst, B., Anderson, A., and Pietsch K. (2008). Validation and collaborative study of a P35S and T-nos duplex real-time PCR screening method to detect genetically modified organisms in food products. *Eur Food Res Technol* 226, 1221–1228. doi:10.1007/s00217-007-0748-z
